# Cellular Responses to Nanoscale Topography Mediated Through the RhoA/ROCK Pathway

**DOI:** 10.1002/smll.202505685

**Published:** 2025-08-18

**Authors:** Erik N. Schaumann, Nam Heon Cho, Teri W. Odom

**Affiliations:** ^1^ Department of Chemistry Northwestern University Evanston IL 60208 United States; ^2^ Department of Materials Science and Engineering Northwestern University Evanston IL 60208 United States

**Keywords:** cytoskeleton, nanoparticles, nanoscale curvature, nanotopography, RhoA

## Abstract

Nanotopography exhibits strong effects on cellular properties such as cytoskeletal organization and endocytosis. Responses to topographical cues can propagate into cell‐scale characteristics like cortical stiffness as well as systemic effects like inflammation and implant rejection; however, the biological pathways governing these effects remain comparatively unknown. Here we show how the RhoA/ROCK pathway can regulate responses to nanotopographical features vis‐à‐vis cellular tension. 2D arrays of hemispherical gold nanoparticles are combined with epithelial cells to determine how up‐ and down‐regulating RhoA activity influences cellular responses. It is found that higher RhoA activity correlates with increased tension and decreased membrane conformality, actin reorganization, and endocytosis. Also, increased tension produces large focal adhesions in cells not typically observed from substrate patterns. Conversely, reducing tension by lowering RhoA activity results in increased membrane conformality around the nanoparticles as well as actin and endocytosis colocalization with nanoparticle sites. This study provides a critical connection between biomolecular regulators of cell mechanics, in particular RhoA, and cellular responses to nanoscale topographical features.

## Graphical abstract

The most common ways that cells respond to nanoscale patterns include cytoskeletal reorganization, reduction in focal adhesion density, and increased endocytosis. Here RhoA is identified as a single biological pathway that can unify cellular responses to nanotopography. This work describes an important way by which nanomaterials may be engineered to produce desired outcomes in cells.

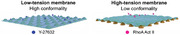

## 1 Introduction

The interface between nanostructures and cells is important for biomedical applications since mechanical properties of the local environment, such as shape and stiffness, transduce into cellular behavior through distinct pathways.^[^ ^1^, ^2^, ^3^ ^]^ This transduction mechanism can have dominant effects in diverse areas, from cancer biology to tissue engineering to implantable devices.^[^ ^4^, ^5^ ^]^ For example, micron‐sized patterned topographical cues in the form of pillars can direct cancer cell migration to regions of decreased feature densities (topotaxis) by mechanosensitive pathways.^[^ ^6^, ^7^ ^]^ Moreover, nano‐topography can induce cellular responses equivalent to that from substrate stiffness, such as cells grown on quartz nanopillars displaying morphological and cortical stiffnesses similar to cells on flat elastomeric surfaces.^[^ ^8^ ^]^ This reproduction in cell characteristics on substrates that are either flexible and unpatterned or rigid and patterned suggests that a common biological pathway can influence both stiffness sensing and topographical responses. We hypothesize that one candidate is the Rho family of GTPases since they regulate many physical properties of cells such as shape and motion.^[^ ^9^ ^]^ Also, because Rho‐GTPases are upstream of many processes involved in curvature sensing, RhoA, which primarily functions to maintain tension in the cytoskeleton,^[^ ^10^, ^11^ ^]^ may serve as a regulator of nanotopographical responses.

Actin rearrangement to form branched networks is a common cellular response to topography from inorganic nanopillar and nanoneedle structures. This pathway was found to proceed through three steps involving the recruitment of the actin‐related protein 2/3 (Arp2/3) complex,^[^ ^12^, ^13^, ^14^ ^]^ an actin nucleator that specifically leads to branched actin, which is the final cytoskeletal component of curvature sensing.^[^ ^15^ ^]^ Nanoridges can also induce actin reorganization by providing directional cues that result in cell elongation and migration.^[^ ^16^ ^]^ The corners of ridges present curved surfaces similar to pillars for actin localization, and actin flows from the periphery to the nucleus (retrograde flow) and orients along the grooves.^[^ ^17^, ^18^ ^]^ This effect was observed for patterned substrates with both short (100 nm) and tall (1 µm) ridges; moreover, taller features induce stronger cellular responses for nanopillars and nanoridges.^[^ ^19^, ^20^ ^]^ Actin organization is also affected by RhoA, which promotes stress fiber assembly;^[^ ^21^ ^]^ therefore, RhoA is antagonistic with branched actin network assembly associated with cellular interfaces and nanotopography since stress fibers are composed of actin filament bundles.

In addition, nanopillar patterns have been shown to recruit endocytosis‐related proteins clathrin and dynamin because they are associated with membrane curvature. The localization of endocytic proteins at micron‐tall nanopillars indicates that the formation and scission of clathrin‐coated pits, key intermediate structures in receptor‐mediated endocytosis, are amplified at sites of nanotopography.^[^ ^22^, ^23^ ^]^ In contrast, short (200 – 250 nm) nanopillars and nanoridges have exhibited contradictory endocytosis responses: some assays showed increased internalization but others did not.^[^ ^24^ ^]^ Together these reports suggest a link between topographical curvature and endocytosis across length scales and that membrane conformation plays an important role. RhoA/ROCK controls endocytosis by inhibiting the formation of clathrin‐coated pits and by affecting how cells induce curvature changes through membrane invagination.^[^ ^25^ ^]^

Another cellular response to nanotopography is changes in the morphology and distribution of focal adhesions^[^ ^8^, ^15^, ^26^ ^]^ that act as contacts between the cytoskeleton and extracellular environment. Structures with heights greater than 1 µm preclude normal focal adhesion formation and promote integrin endocytosis,^[^ ^8^, ^14^, ^15^ ^]^ resulting in curved focal adhesions around the sides of pillars.^[^ ^27^ ^]^ With decreased feature heights in the range of 200 nm, focal adhesions form over their entire structure but at reduced density.^[^ ^26^ ^]^ Mature focal adhesions, marked by the presence of the protein vinculin, require a minimum of applied cytoskeletal tension and retrograde flow at the terminus of stress fibers.^[^ ^28^, ^29^ ^]^ As such, the RhoA pathway together with its downstream component ROCK have been identified as crucial regulators of focal adhesion prevalence and maturation.^[^ ^30^ ^]^

Several open questions remain regarding how nanotopographical structures are transduced into cellular responses. Most work has focused on tall, micron‐sized features, with the assumption that aspect ratio is an important parameter.^[^ ^19^, ^31^ ^]^ However, other reports have found that low‐aspect ratio ridges^[^ ^23^ ^]^ and even vesicles^[^ ^32^ ^]^ can induce responses such as curvature‐sensing protein recruitment and enhanced endocytosis. Although the end results of nanoscale curvature sensing are well‐defined, i.e., branched actin formation, increased endocytosis, and reduced focal adhesion density, the underlying mechanobiological pathways are less clear. Since the RhoA/ROCK pathway affects each of these end points separately, and in settings without patterns, this pathway likely plays a common role in regulating responses to topographical stimuli as well.

Here we show that RhoA can regulate curvature sensing responses on patterned arrays of low‐aspect ratio nanoparticles. Gold nanoparticle sites can support Arp2/3‐mediated curvature sensing, with cytoskeletal reorganization depending on RhoA/ROCK activity. Increased RhoA treatment inhibits cellular responses while decreased ROCK activity promotes them. Similarly, decreased tension in cell membranes resulted in increased prevalence of endocytic proteins and colocalization of focal adhesions with array sites; increased tension had the opposite effect. Our results are consistent with a model where tension regulates conformality of the membrane around nanoparticles, which in turn affects the end points of curvature sensing pathways. Our study has identified a single biological pathway relating different cellular responses to nanotopography as well as to each other under the regulatory effects of RhoA. Understanding how cellular downstream effects can be correlated with specific pathways and nanopatterns will open prospects to engineer surfaces for desired biological outcomes.

## 2 Results

### 2.1 Cells Form Conformal Interfaces with Hemispherical Nanoparticle Arrays

**Figure 1:**
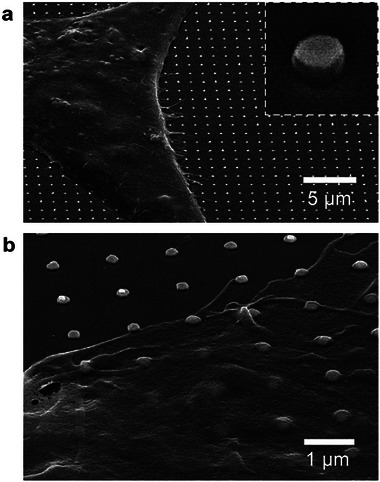
SKOV3 cells form nano‐bio interfaces around gold nanoparticles. a) Tilted SEM image (45°) of a single SKOV3 cell on a gold nanoparticle array. Inset (500 × 500 nm) shows a zoom of a single nanoparticle. b) Tilted SEM image (45°) showing cell membrane wrapping around nanoparticles.

To determine the impact of RhoA on curvature‐related cellular responses, we first fabricated arrays of hemispherical gold nanoparticles with heights = 80 nm and diameters = 150 nm and then cultured epithelial cells on the nanopatterns (Figure S1, Supporting Information). Scanning electron microscopy (SEM) images revealed conformal cell growth on gold nanoarrays on quartz substrates (**Figure** **1**a), with filopodial protrusions interacting directly with nanoparticles. We selected the sizes of nanoparticles based on predicted curvature sensing responses^[^ ^33^ ^]^ and gold as the nanostructured material because of its biocompatibility and ability for covalent functionalization via gold‐thiol chemistry. Quartz was used as the substrate material for ease of patterning since there is no induced cytotoxicity unless fractured.^[^ ^34^ ^]^ Moreover, curvature sensing has been shown to be insensitive to material properties, likely since proteins initiating these responses are intracellular.^[^ ^23^, ^35^, ^36^ ^]^ We focused on the ovarian cancer cell line SKOV3 as a model system to evaluate topographical responses since these cells are adherent and express upregulated receptors that can be targeted.^[^ ^37^, ^38^ ^]^ Compared to cells grown on plain glass substrates, cells on arrays were more spread out and less regular in shape,^[^ ^39^ ^]^ which is consistent with increased cell motility (Figure S2, Supporting Information). Figure 1b indicates that cells grown on gold nanoparticle arrays also showed membrane wrapping around some individual nanoparticles but in an ill‐defined manner.

**Figure 2:**
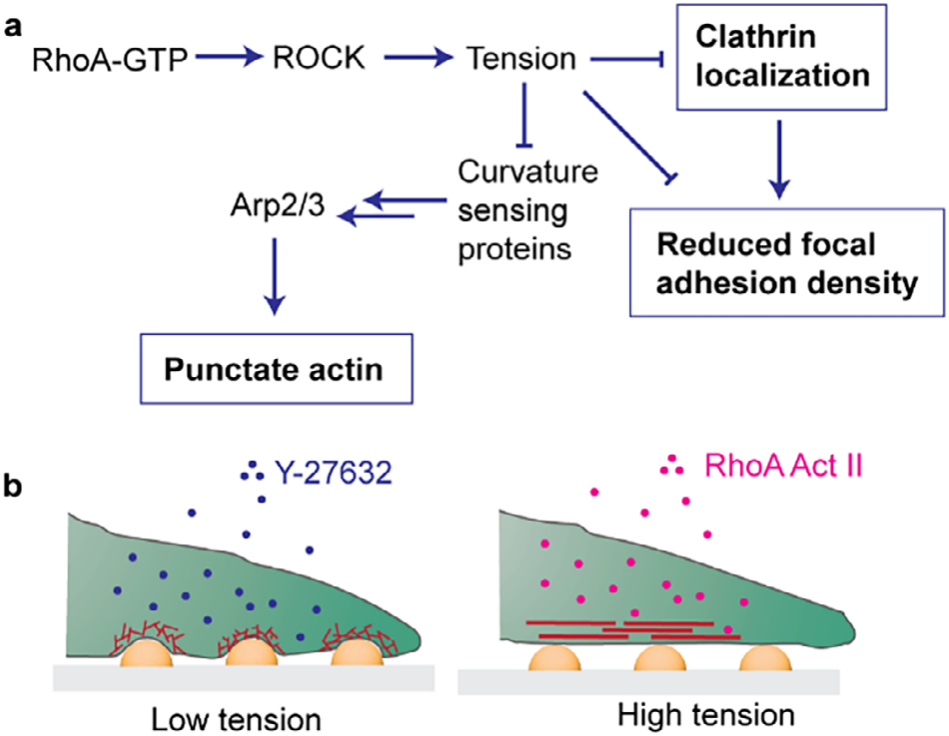
Rho‐mediated cytoskeletal tension determines curvature at nanotopographical sites. a) Diagram of RhoA/ROCK pathway related to curvature sensing without topographical stimuli. Boxes indicate biological endpoints tested. b) Scheme of how actin cytoskeleton in cells reorganizes around topographical nanofeatures under conditions of low and high tension.

**Figure** **2**a summarizes how, without nanotopographical stimuli, the RhoA/ROCK pathway can manipulate tension and induce different cellular responses. First, the GTP‐bound form of RhoA can phosphorylate ROCK, resulting in increased tension in cells. This increased tension leads to a decrease in the ability of curvature‐sensing proteins to localize to membrane invaginations, which then prevents the Arp2/3‐mediated pathway from forming puncta of branched actin. In addition, higher tension decreases clathrin localization around curved membrane sites.^[^ ^23^ ^]^ Focal adhesions also respond to higher tension with increased density and maturation, but a side effect of clathrin enrichment is the lowering of focal adhesion density by enhancing integrin endocytosis; hence, increased tension also indirectly raises focal adhesion numbers.

To identify how these processes change in the context of nanoscale features, we modulated the pathway using the common ROCK inhibitor compound Y‐27632 and a RhoA activator reagent (RhoA Act II) that leads to a persistently GTP‐bound, active form of Rho (Figure 2b). Y‐27632 decreases the overall contractility of cells, which disfavors stress fiber production, while RhoA Act II increases contractility and promotes fiber formation. Under low‐tension conditions produced in response to Y‐27632, the cell membrane conforms around topographical features and facilitates curvature sensing and branched actin network formation. Conversely, under high tension conditions produced in response to RhoA Act II, the membrane is not conformal to the nanoparticles, and curvature sensing is negligible.

### 2.2 RhoA Controls Cytoskeletal Remodeling Around Nanoparticles

**Figure 3:**
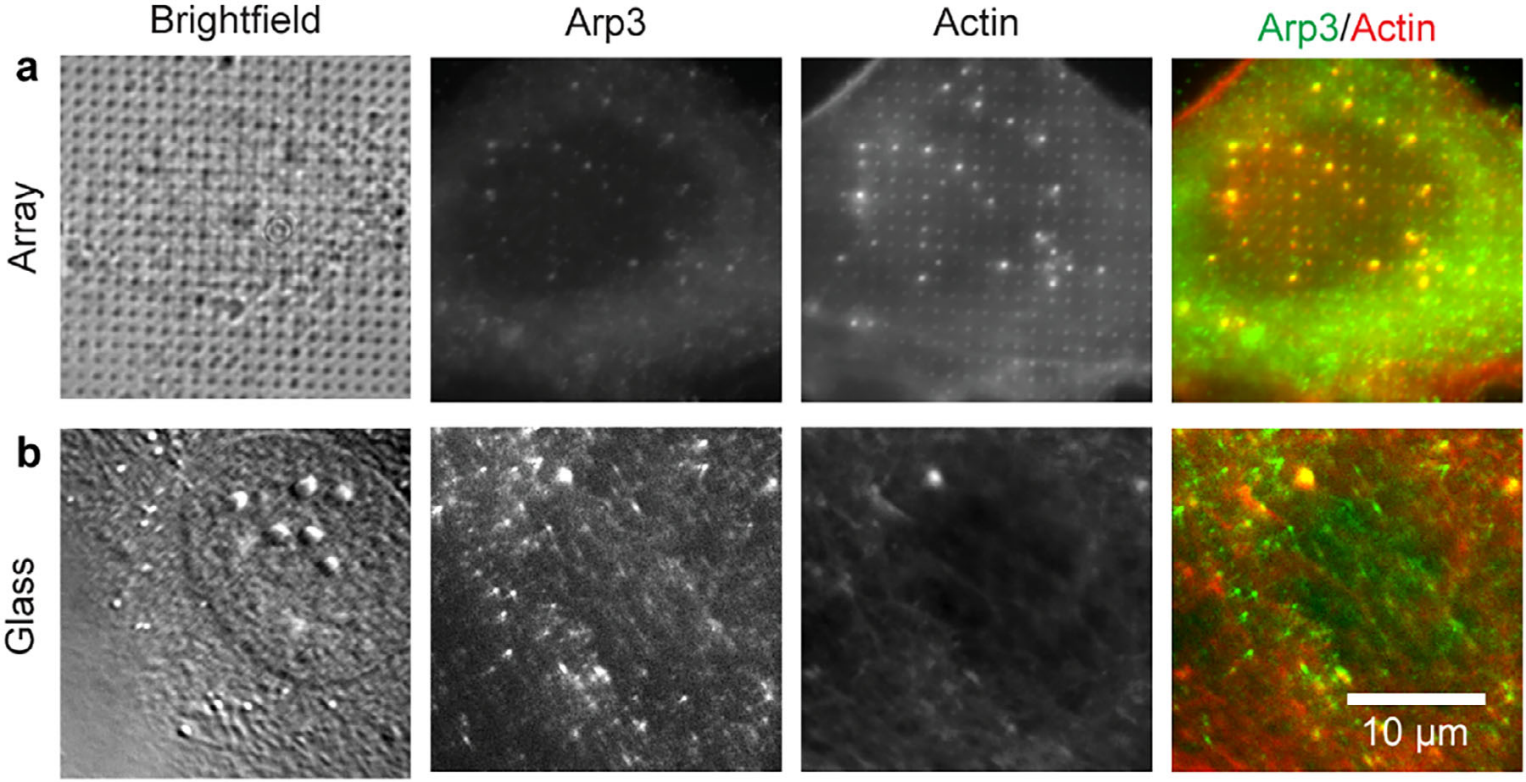
Arrays of hemispherical particles induce accumulation of both actin and Arp3 at nanoparticles. a) Bright puncta in the Arp3 and actin channels under immunofluorescence show cytoskeletal reorganization around array sites. b) No Arp3/actin puncta are observed in cells grown on unpatterned glass. All images are taken at the basal surface of cell.

**Figure** **3**a shows how nanoparticle arrays can result in co‐localization of actin and Arp3 at the array sites. Brightfield microscopy resolved the 2D square array pattern along with cellular features, and immunostaining was used to visualize the proteins involved in curvature sensing. Puncta of Arp3, a component of Arp2/3 that serves as a step in cytoskeletal reorganization, and puncta of actin are visible on the gold nanoparticle sites to varying degrees of brightness. The brightest Arp3 and actin puncta are colocalized, consistent with the formation of branched actin networks around array sites.^[^ ^15^ ^]^ In contrast, on unpatterned glass, cells displayed few colocalized structures (Figure 3b). While the Arp3 channel displays occasional puncta, they are not associated with equivalent puncta of actin. Furthermore, the actin channel displays primarily thin fibrils. We verified curvature sensing by correlating the Arp3 and actin channels in regions with high densities of puncta and determined that they displayed higher correlation than regions on glass (Figure S3, Supporting Information). These colocalized puncta are not uniform across the entire cell and also vary in brightness, which could reflect two features of the puncta. The first is the dynamic nature of the structures that have shown to be in a constant state of assembly and disassembly; thus, at different time points, they appear randomly throughout the cell.^[^ ^15^ ^]^ The second is that since RhoA regulates curvature sensing, the scattered distribution of puncta may also result from the uneven distribution of RhoA within cells.^[^ ^40^ ^]^ We also tested whether array pitch affected the cellular response and found the same constellation of Arp3‐colocalized actin puncta with a smaller pitch (*a*_0_ = 600 nm) (Figure S4, Supporting Information).

**Figure 4:**
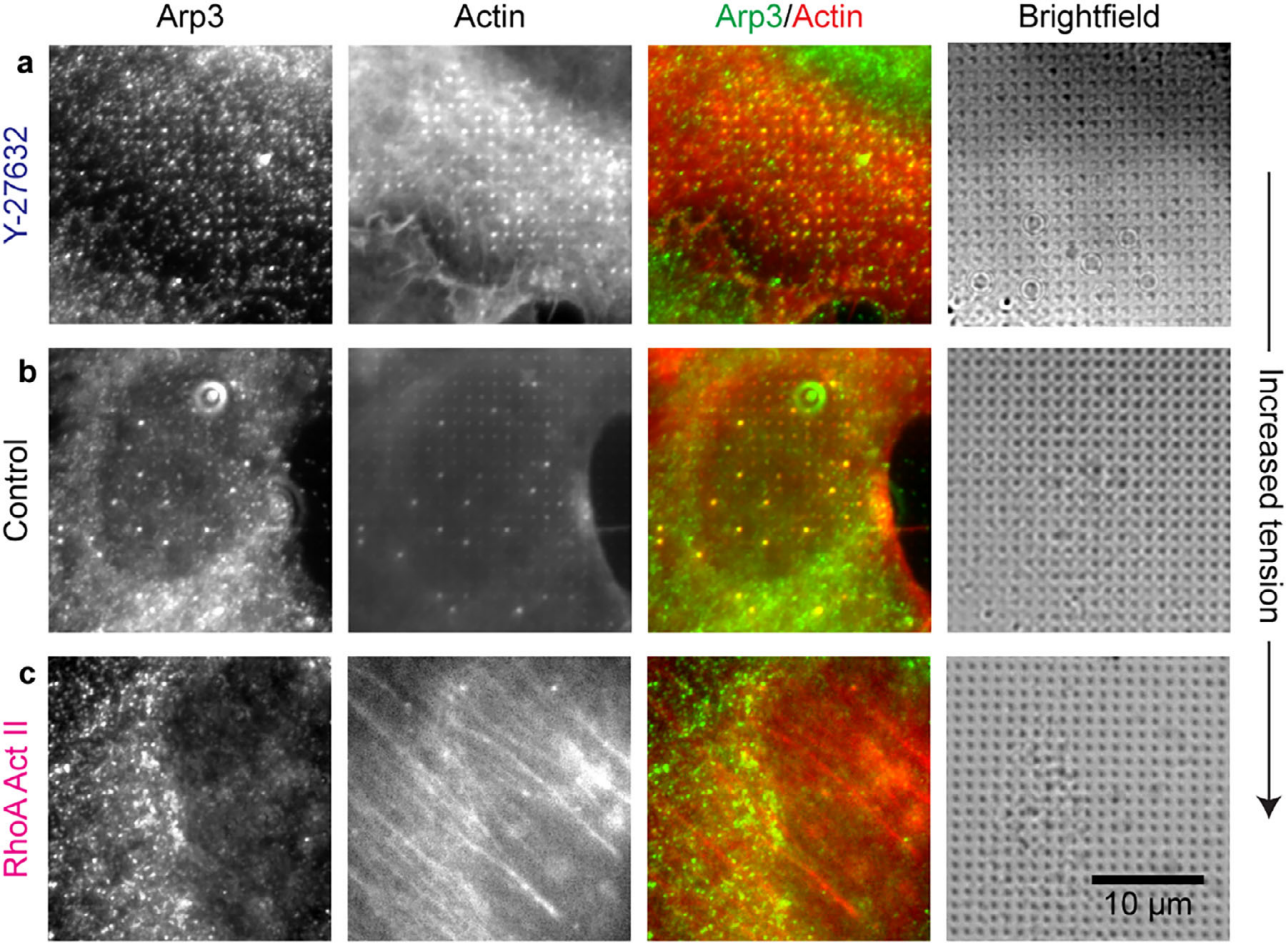
RhoA pathway regulates curvature sensing response. a) Treatment with Y‐27632 to lower cytoskeletal tension yields increased prevalence of Arp3/actin puncta in the relevant immunofluorescence channels, and the underlying array structure can be observed in both channels. b) Control experiment without modifying tension shows that puncta are present but with limited co‐localization. c) Increased tension induced by RhoA Act II results in the elimination of puncta and only stress fibers are formed.

Since the nanoparticle arrays can induce cytoskeletal remodeling, we next investigated the role of the RhoA/ROCK pathway in modulating this response by treating with Y‐27632 and RhoA Act II. We found that lowering the tension with a standard dosage^[^ ^41^ ^]^ of Y‐27632 (20 µM) resulted in increased frequency of Arp3 and actin puncta. Across the imaged cell area, nearly a one‐to‐one correspondence between nanoparticles and associated puncta was present in both actin and Arp3 channels (**Figure** **4**a). The upper‐right and lower‐left corners of the image show the nucleus and a cell‐cell junction, respectively. Since membrane conformality appears primarily at the edges of cells (Figure S2, Supporting Information), the puncta are not as prevalent in these areas. Otherwise, the high frequency of puncta co‐localization was in marked contrast with untreated (control) cells, which only showed constellation‐like distributions of Arp3/actin puncta (Figure 4b), as expected and like Figure 2b. Although several faint actin puncta appear colocalized with Arp3 in a grid‐like pattern, these spots may represent puncta undergoing assembly or disassembly.^[^ ^15^ ^]^

With the application of RhoA Act II (1 µg mL^−1^), we observed a near‐total abrogation of puncta and a noticeably higher density of stress fibers (Figure 4c). The overall structuring of the actin channel also reveals important characteristics, where under low‐tension conditions, fast Fourier transform images of actin show a 2D periodic structure (Figure S5, Supporting Information). Under control conditions, the observation of periodicity in the actin channel is significantly reduced, and under increased tension, none is present. In addition, we tested for the extent of colocalization between actin and nanoparticle sites by measuring the correlation between the actin and brightfield channels; cells treated with Y‐27632 had significantly more correlation (Figure S6, Supporting Information). Overall, these results are consistent with a model in which higher tension associated with increased RhoA activity leads to decreased conformality of the membrane around array sites as well as higher stress fiber production (Figure 2b; Figure S7, Supporting Information).

### 2.3 Focal Adhesion Density on Arrays is Regulated by RhoA Activity

**Figure 5:**
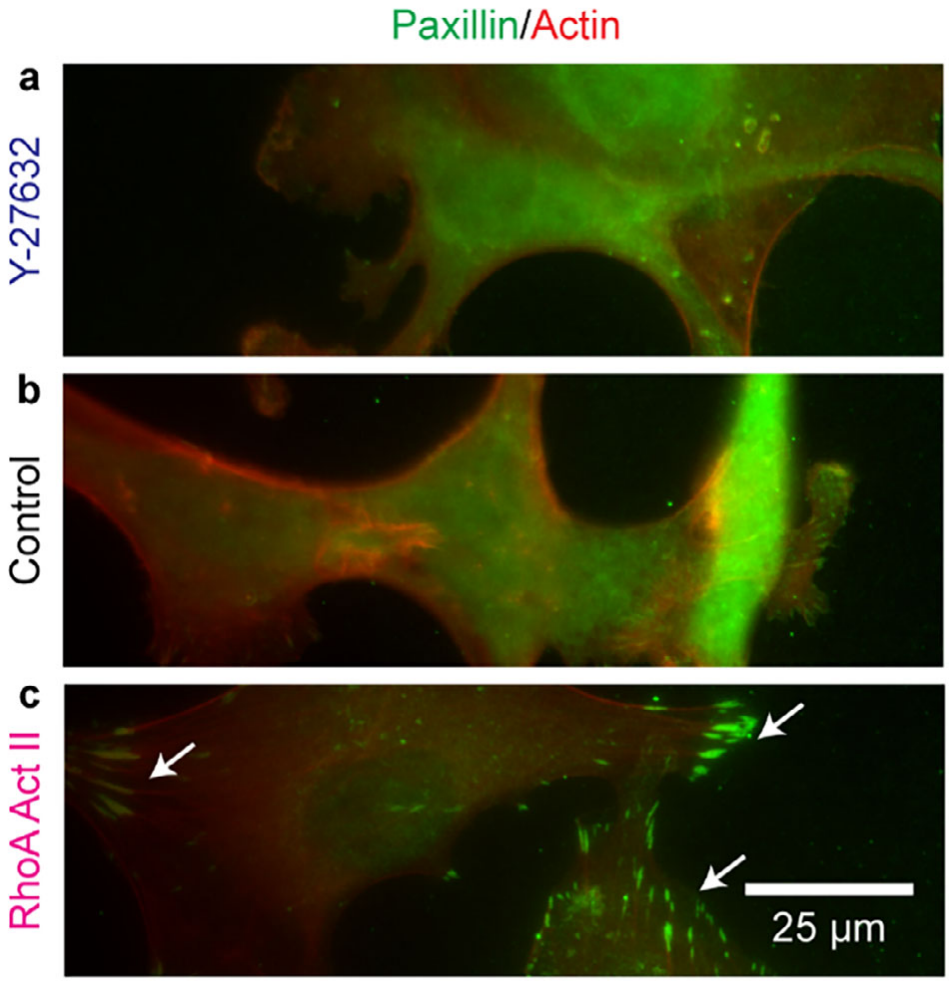
RhoA Act II can recover focal adhesion morphology in cells grown on patterned structures. Focal adhesion protein expression indicated by paxillin immunofluorescence is predominantly cytosolic in cells grown on gold nanoparticle arrays (a) with ROCK or (b) without ROCK inhibition. However, increased tension (c) recovers the plaque morphology that is common for focal adhesions on unpatterned hard substrates. Arrows indicate focal adhesion plaques.

Focal adhesions are responsive to both RhoA activity and topography. Nanostructure patterns increase integrin endocytosis, which reduces focal adhesion density, and focal adhesion maturation depends on tension and local actin architecture, two factors that are increased by RhoA. To visualize focal adhesion morphology, we immunostained for the nascent focal adhesion protein paxillin. Cells on nanoparticle arrays treated with Y‐27632 displayed a decrease in typical mature plaque presentation as well as increased paxillin in the cytosol (**Figure** **5**a). Untreated cells grown on gold nanoparticles also exhibited limited numbers of focal adhesion plaques and primarily showed cytosolic paxillin (Figure 5b). Cells on unpatterned glass exhibited focal adhesion plaques at the ends of stress fibers and that were considerably larger (Figure S8, Supporting Information). Since both Y‐27632‐treated and untreated conditions lack stress fibers, there is no specific association between regions of paxillin density and actin signals. We note that actin puncta cannot be resolved at the lower magnification (20x) of these images, which we used to capture the paxillin localized to cellular interiors under Y‐27632 and control conditions. In previous studies using tall features, the reduction in focal adhesion density was linked to increased endocytosis of integrins.^[^ ^8^ ^]^ Figure 5c shows how treatment with RhoA Act II could recover the focal adhesion plaque morphology usually observed from cells on flat substrates. Quantification of focal adhesion sizes under different levels of RhoA activity is summarized in Figure S9 (Supporting Information). With increased RhoA activity, cells grown on arrays can recover focal adhesion sizes observed on unpatterned glass. Hence, cell migration speed should be faster under RhoA activation since larger focal adhesions strongly correlate with higher motility.^[^ ^42^ ^]^

**Figure 6:**
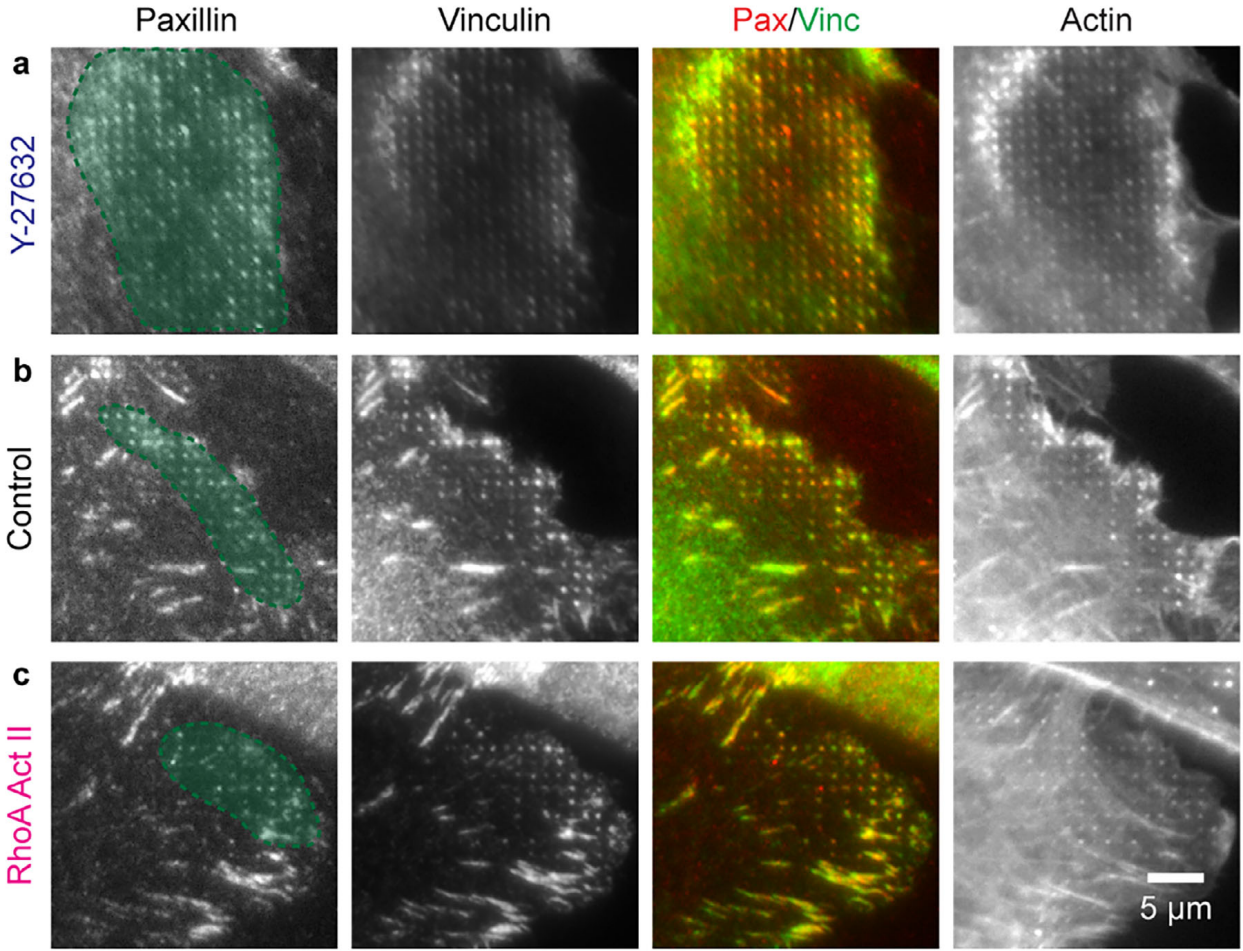
RhoA‐induced tension affects distribution but not maturation state of nanoparticle‐associated focal adhesions. a) With ROCK inhibition, focal adhesions colocalized with array sites (green shading) are prevalent across the spread area of each cell as shown by immunofluorescence. Additionally, vinculin signal overlaps with paxillin indicating that the array‐associated focal adhesions are mature. b) With no pharmacological treatment, there are still patches of mature focal adhesions, but they are smaller compared to the Y‐27632 case. c) With RhoA activation, the patches of mature focal adhesions were similar in size to the control.

To determine how the distribution of nascent and mature focal adhesions, respectively, varied with respect to RhoA activity, we next co‐immunostained for paxillin and vinculin. **Figure** **6**a shows that under low‐tension conditions (Y‐27632 treatment), the most striking features were large, contiguous patches of 2D array structures; there was a one‐to‐one correspondence between gold nanoparticle sites and focal adhesions. Although these patches were still present at increased levels of tension, they tended to be smaller (Figure 6b,c). To compare quantitatively the extent of focal adhesion formation on the nanoparticle sites, we measured the patch sizes and found that Y‐27632‐treated cells had the largest areas, with control and RhoA Act II‐treated cells displaying correspondingly smaller patches of similar sizes (Figure S10, Supporting Information). Patch size dependence under lower tension suggests that recruitment of focal adhesion proteins to array sites depends on membrane conformality around the nanoparticles.

Surprisingly, we observed that adhesion maturation at array sites was not affected by the level of RhoA activity, and array‐associated paxillin puncta were uniformly colocalized with equivalent vinculin puncta. Hence, array‐associated focal adhesions can recruit vinculin without being the terminus of stress fibers. Moreover, the tension and actin architecture at array sites must be capable of supporting focal adhesion maturation.^[^ ^29^ ^]^ This finding is consistent with reports that Y‐27632 did not impair growth on its own (assuming actin bundling supported by α‐actinin and mDia1 are at focal adhesions).^[^ ^29^ ^]^ Although mDia1 does not preferentially localize to array features,^[^ ^15^ ^]^ our results suggest that both mDia1 and α‐actinin are present around nanoparticles in sufficient quantities to enable vinculin association with focal adhesions. That focal adhesion growth occurs with Y‐27632 treatment combined with topographical features, which together should prevent this process, is thus significant and demonstrates the robustness of maturation at nanoparticle sites.

### 2.4 RhoA Controls the Extent of Endocytic Protein Localization on Arrays

**Figure 7:**
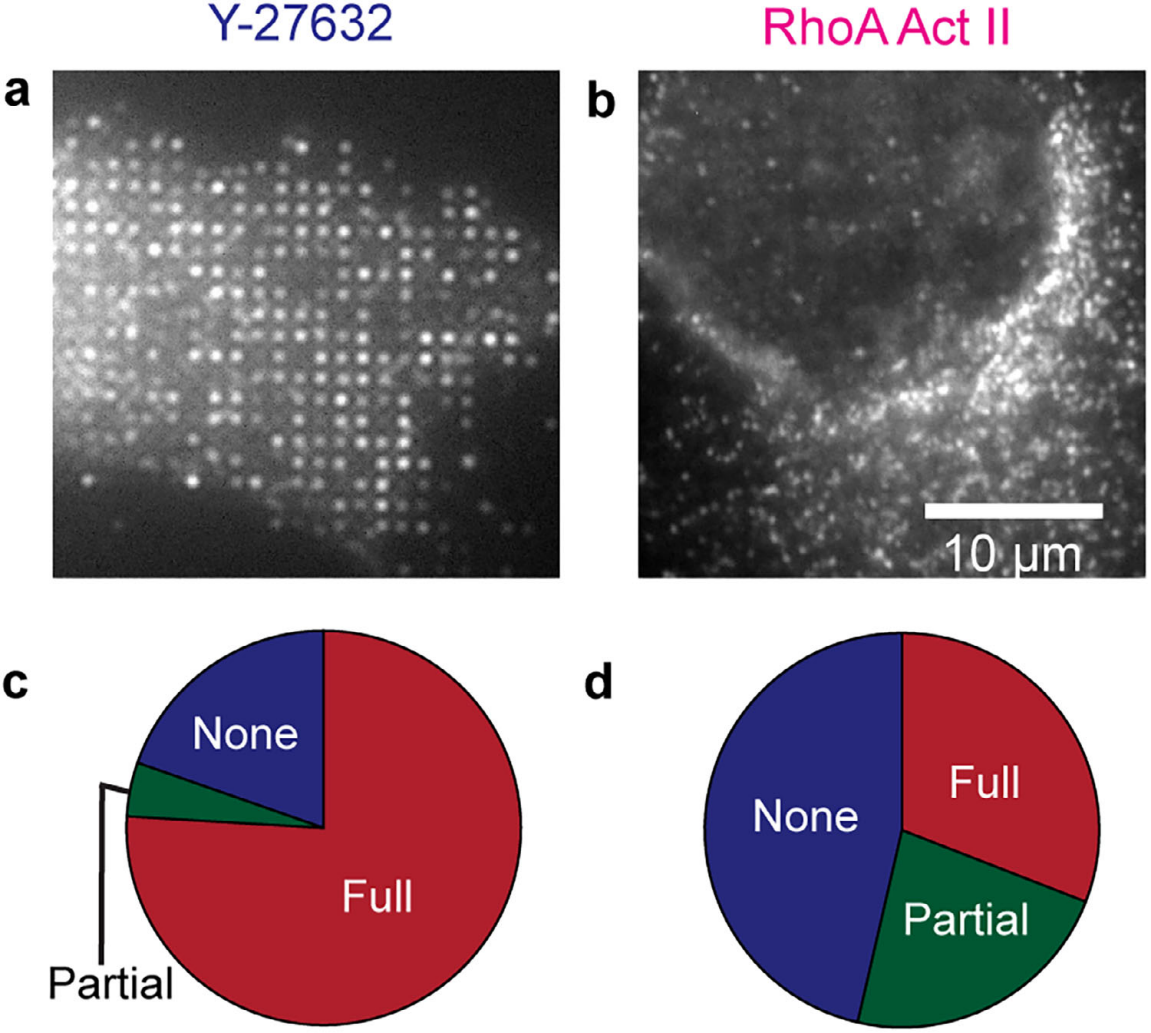
RhoA regulates response to topography‐mediated endocytosis. a) Under Y‐27632 treatment, clathrin‐coated pits visible under immunofluorescence become ubiquitous across the cell spread area, and the underlying array pattern is apparent in the clathrin channel. b) Under Rho Act II treatment, clathrin is less prevalent, and the underlying array pattern is less commonly observed. c,d) Pie charts indicating the extent to which array patterning is visible in the clathrin channel under low‐ and high‐tension conditions.

Having established that the RhoA/ROCK pathway affects focal adhesion properties on arrays, we next focused on internalization since integrin endocytosis modulates focal adhesion density.^[^ ^8^ ^]^ Since clathrin‐mediated endocytosis can be enhanced both by nanostructures^[^ ^22^ ^]^ and ROCK inhibition,^[^ ^43^ ^]^ we tested whether the additive effects of arrays and RhoA/ROCK modulation could control this enrichment. Under ROCK‐inhibited, low‐tension conditions, clathrin staining revealed puncta across the spread area of the cell that followed the array structure (**Figure** **7**a). In contrast, while the addition of RhoA Act II did not completely eliminate clathrin‐coated pit formation at array sites (Figure 7b), areas with visible array patterning was reduced.

Due to variations in signal intensity that precluded automated counting of puncta, we manually defined masks to classify the total spread area of each cell into three categories: full patterning (one‐to‐one correspondence of nanoparticles with visible clathrin puncta); partial patterning (only some array sites had associated clathrin puncta); and no patterning. Figure 7c shows that full patterning was the predominant pattern for Y‐27632‐treated cells (75.9%), followed by no patterning (19.6%) and partial patterning (4.4%). Meanwhile, RhoA Act II‐treated cells (Figure 7d) displayed a more balanced distribution between full patterning (30.9%), partial patterning (22.7%), and no patterning (46.4%). This difference in clathrin‐coated pit formation indicates that the RhoA/ROCK pathway has an antagonistic relationship with clathrin‐mediated endocytosis. Low‐tension conditions produce a conformal membrane facilitates clathrin‐coated pit formation at the nanoparticles, while high tension inhibits membrane conformation and reduces clathrin localization to array sites. Since integrin endocytosis proceeds through clathrin‐mediated pathways,^[^ ^44^ ^]^ our observations suggest that RhoA activity can also affect integrin endocytosis on nanostructures.

## 3 Conclusion

This work has shown how the RhoA pathway can be linked to curvature sensing at the nanoscale and serve as an important regulator of nanoscale topographical sensing. We found that RhoA activity has an inverse relationship with the extent of cytoskeletal remodeling, focal adhesion density changes, and clathrin‐mediated endocytosis. Under low cytoskeletal tension, the cellular membrane conforms around nanoparticle sites to initiate a curvature sensing response. Conversely, under high‐tension conditions, there is less conformality, and there are no curvature sensing proteins to initiate topographical sensing pathways. Hence, we have unified the three most notable responses to nanotopography—cytoskeletal reorganization,^[^ ^15^ ^]^ focal adhesion reduction,^[^ ^14^, ^15^ ^]^ and endocytosis enhancement^[^ ^22^ ^]^—under a single pathway. This work provides insight into how curvature sensing can connect to other nanoscale topography effects on processes such as macrophage polarization,^[^ ^45^ ^]^ antibacterial implant surfaces,^[^ ^46^ ^]^ stem cell differentiation,^[^ ^47^ ^]^ and molecular delivery.^[^ ^48^ ^]^

We envision modifying RhoA/ROCK in conjunction with patterned substrates can promote desired effects at different bio‐nano interfaces. For example, previous work on colloidal gold nanoparticles functionalized with single‐stranded DNA aptamers such as AS1411^[^ ^49^ ^]^ and anti‐HER2‐aptamer^[^ ^50^ ^]^ demonstrated targeting of cancer‐associated receptors nucleolin and HER2 on live cell membranes, respectively. We anticipate that future work can evaluate how these nanoconstructs are taken up in detail using nanoparticle arrays and RhoA/ROCK treatment to optimize therapeutic delivery. Furthermore, our work may provide insight into the engineering of 3D scaffolds decorated with nanoparticles to control cellular responses. The effects we observed regarding cell shape parameters and focal adhesions may be relevant for vascularization and organ‐on‐a‐chip designs.^[^ ^51^, ^52^ ^]^ Finally, we anticipate that other Rho‐GTPases, such as Cdc42 and Rac, may serve as regulators for nanotopography.^[^ ^53^ ^]^ Modulating mechanical properties that can also be correlated with biochemical cues promises to open ways to engineer nano‐bio interactions that can both reduce side effects and promote desired health outcomes.

## 4 Experimental Section

### Fabrication of Nanoparticle Arrays

Arrays of gold nanoparticles were fabricated on quartz substrates by a combination of solvent‐assisted nanoscale embossing (SANE)^[^ ^54^ ^]^ and reactive ion etching. Substrates for patterning started with 80 nm of gold deposited on quartz slides by thermal vapor deposition (Nano 35, Kurt J. Lesker) and 10 nm of SiO_2_ produced by atomic layer deposition (Arradiance ALD XT‐P). To create the array patterns, photoresist S1805 was mixed with propylene glycol methyl ether acetate in a volume ratio of 1:16, spin‐cast on the gold/SiO_2_ substrate, and baked at 120 °C. Then, SANE was performed, where a prepatterned PDMS stamp was immersed in *N,N*‐dimethylformamide for 90 s and then placed on the photoresist‐covered substrate for 45 min to mold the photoresist into the desired pattern of photoresist pillars.

For pattern transfer to the substrate, the photoresist pillars were first cleaned using reactive ion etching (RIE) (RIE‐10NR, Samco) with an oxygen plasma (500 sccm O_2_, 20 Pa, 70 W, 30 s). The SiO_2_ dielectric layer was then etched with RIE (200 sccm CHF_3_, 10 sccm Ar, 1.33 Pa, 100 W, 2 min). All remaining photoresist was removed with a second oxygen plasma step (12 min) so that the underlying gold film would undergo uniform etching. The gold was etched using Ar plasma (20 sccm Ar, 2 Pa, 150 W, 8 min), and a second SiO_2_ etch (2 min) removed the remaining dielectric material to reveal arrays of short cylindrical gold nanoparticles. In order to present a constantly curved geometry to the cells, we converted the shapes to hemispheres by thermal annealing (25 sccm Ar, 1.2 Torr, 900 °C, 30 min) in an MTI furnace system (OTF‐1200x). 2D arrays of hemispherical nanoparticles were used for all experiments.

### Cell Culture and Reagents

Human ovarian carcinoma SKOV3 cells were purchased from ATCC and maintained in complete cell medium consisting of McCoy's 5A medium (Gibco) supplemented with 10% fetal bovine serum (Gibco). Cells were maintained at 37 °C and 5% CO_2_ and cultured on T‐25 flasks (Corning). For imaging, cells of passage number less than 20 were plated on unpatterned glass or patterned quartz substrates at a density of 10,000 cells cm^−2^ in a custom designed PDMS (Sylgard^TM^ 184) chamber with interior area 1 cm^2^. Cells were then incubated for 24 h under normal culture conditions before being prepared for immunofluorescence imaging. Y‐27632 (Sigma‐Aldrich) was used at a concentration of 20 µM and a treatment time of 1 h to inhibit ROCK activity in cells. RhoA Activator II (Cytoskeleton) was used at a concentration of 1 µg mL^−1^ and a treatment time of 3 h to increase the RhoA phenotype in cells. We established that treatment with Y‐27632 or RhoA Act II did not alter the overall cell shape (Figure S11, Supporting Information). Drug dosages were selected for their strong predicted effects on cellular responses to nanotopography. We note that reduced dosages may also induce more subtle effects.^[^ ^29^ ^]^

### Immunofluorescence

Cells were fixed in a solution of 4% paraformaldehyde (Electron Microscopy Sciences), 0.15% w/v bovine serum albumin (Sigma‐Aldrich), and 0.5% Triton X‐100 (Sigma‐Aldrich) in phosphate‐buffered saline (Corning). The staining solution was composed of 0.15% w/v bovine serum albumin and 0.5% Triton X‐100 in phosphate‐buffered saline. The fixing solution was warmed in a 37 °C water bath for 1 h prior to cell fixation, which was conducted for 15 min at room temperature. Samples were then washed 3x for 5 min each with phosphate‐buffered saline before being transferred to primary antibody solutions. Samples were kept at 4 °C overnight in primary antibody before being returned to room temperature and washed 3x for 5 min each with phosphate‐buffered saline. Samples were then incubated in secondary antibody at room temperature for 1 h before a final wash step 3x for 5 min each in phosphate‐buffered saline. Glass substrates were mounted onto glass slides (VWR), while annealed arrays were mounted on 64 × 20 mm glass coverslips (VWR) using ProLong^TM^ Glass Antifade mountant (Thermo‐Fisher) for 24 h prior to imaging. All images were collected at the basal surface of the cells, coinciding with the focal plane of the arrays on samples where arrays were present. All immunofluorescence images depicted are representative from n = 12 – 42 fields of view collected from each sample. Experiments were conducted in duplicate.

### Fluorescent Antibodies

We used mouse anti‐Arp3 at a dilution of 1:200 (Abcam), rabbit anti‐paxillin at a dilution of 1:100 (Proteintech), mouse anti‐vinculin at a dilution of 1:100 (Thermo‐Fisher), goat anti‐mouse AlexaFluor 488 at a dilution of 1:800 (Abcam), and donkey anti‐rabbit AlexaFluor 568 at a dilution of 1:200 (Abcam). Additional immunofluorescence reagents included phalloidin‐iFluor 647 (Abcam) at a dilution of 1:800 from the recommended stock concentration, and Hoescht stain at a 1:2000 dilution.

### Scanning Electron Microscopy (SEM) Analysis

To prepare samples for SEM imaging, we followed previously published methods.^[^ ^55^ ^]^ First, cells were fixed in a solution of 2.5% glutaraldehyde (Thermo‐Fisher) for 2 h at 4° C. The samples then underwent secondary fixation with 1% osmium tetroxide for 1 h at room temperature and were protected from light. Fixed cells were rinsed three times with PBS for 5 min each round and then dehydrated using successively higher concentrations of ethanol. For dehydration while preserving the cell morphology, the samples were immersed sequentially in 10%, 30%, 50%, 70%, 90%, and twice at 100% ethanol in water for 5 min in each round. Samples were then sputter coated with 5 nm of Au/Pd before being transferred to a Hitachi SU8030. Images were acquired using an acceleration voltage of 10 kV and a current of 20 µA.

## Conflict of Interest

The authors declare no conflict of interest.

## Supporting information



Supporting Information

## Data Availability

The data that support the findings of this study are available from the corresponding author upon reasonable request.
