# Supplementary material for: Cellular Responses to Nanoscale Topography Mediated Through the RhoA/ROCK Pathway
Source: Small. 2025 Aug 18;21(40):e05685. doi: 10.1002/smll.202505685 (PMC12413853; doi:10.1002/smll.202505685)
Supplement: Supplementary file 1 — Supporting Information [file SMLL-21-e05685-s001.docx]

Supporting Information

**Cellular responses to nanoscale topography mediated through the RhoA/ROCK pathway**

*Erik N. Schaumann,^1^ Nam Heon Cho,^1^ and Teri W. Odom*^,1,2^*

^1^Department of Chemistry and ^2^Department of Materials Science and Engineering, Northwestern University, Evanston, Illinois 60208, United States

*Corresponding author: todom@northwestern.edu

Table of Contents

[Supplementary Methods 25](#_Toc204441073)

[Fabrication of annealed gold nanoparticle arrays 26](#_Toc204441074)

[Cell shape is less regular on nanoparticle arrays than on glass 27](#_Toc204441075)

[Correlation between actin and Arp3 is increased on nanoparticle arrays 28](#_Toc204441076)

[Cell shape and curvature sensing remain consistent on arrays with *a*_0_ = 600 nm 29](#_Toc204441077)

[Periodicity of actin puncta decreases with increasing tension 30](#_Toc204441078)

[Treatment with Y-27632 increases correlation between actin and nanoparticle signals 31](#_Toc204441079)

[Stress fiber coverage shows increased tension with increased RhoA activity 32](#_Toc204441080)

[Focal adhesions of cells on glass adopt large plaque morphologies 33](#_Toc204441081)

[Focal adhesions on arrays are larger with higher RhoA activity 34](#_Toc204441082)

[Contiguous patches of array-patterned paxillin are larger with reduced RhoA activity 35](#_Toc204441083)

[Treament with Y-27632 or RhoA Act II does not affect cell shape 36](#_Toc204441084)

[Supporting Information References 37](#_Toc204441085)

# Supplementary Methods

*Actin and nanoparticle correlation measurements*

To establish colocalization between actin and nanoparticle sites, we measured correlation between the actin channel and the brightfield channel, in which the nanoparticles are visible. Since the brightfield channel also shows cellular features such as nucleoli, we selected regions of interest corresponding to locations that only showed array sites. We equalized the contrast across the regions of interest by using CLAHE (available by default in the FIJI distribution of ImageJ) using a blocksize of 19, 256 histogram bins, and a maximum slope of 3. To correct for chromatic aberration causing misalignment between the actin puncta and nanoparticle sites, we registered the images using the StackReg plugin for ImageJ. Last, we measured the Pearson’s correlation coefficient using the Coloc 2 function available in FIJI.

*Focal adhesion size quantification*

To measure the sizes of focal adhesions, we adopted a protocol from the literature.^[15]^ First, we subtracted the background of a paxillin channel image using the default ImageJ function, which works through a rolling ball algorithm with a radius of 25 pixels. Next, we applied CLAHE using a blocksize of 19, 256 histogram bins, and a maximum slope of 3. We then used the exponentiation function to remove low-brightness objects, and applied a threshold using the mean value of the image to generate a mask. Finally, we used the Analyze Particles function to determine the area of each focal adhesion.

# Fabrication of annealed gold nanoparticle arrays

**Figure S1a** shows the fabrication scheme to prepare two-dimensional arrays of hemispherical gold nanoparticles. First, quartz substrates were coated with 80 nm of gold, 15 nm of SiO_2_, and a thin layer of S1805 photoresist. Then, SANE was performed using a polydimethylsiloxane mask soaked in DMF for 90 s to produce photoresist patterns. Successive steps of reactive ion etching (RIE) then transferred these patterns into arrays of cylindrical gold nanoparticles (**Figure S1b**). The steps included: (1) an oxygen-plasma etch to tune the width of patterned photoresist columns; (2) hard mask etch with CHF_3_ and Ar to remove the dielectric from the unpatterned regions; (3) an oxygen plasma to remove residual photoresist; (4) an argon plasma to etch the exposed gold film; (5) hard mask etch again with CHF_3_ and Ar to remove dielectric from patterned nanoparticle tops; and (6) a final oxygen plasma for cleanup. Thermal annealing at 900 °C for 30 min was then carried out to produce arrays of hemispherical gold nanoparticles (**Figure S1c**) ready for cell culturing. While all arrays used for cellular studies had a pitch of 1200 nm (Figure 1), to demonstrate the flexibility of this method, this figure depicts arrays with a pitch of 600 nm.


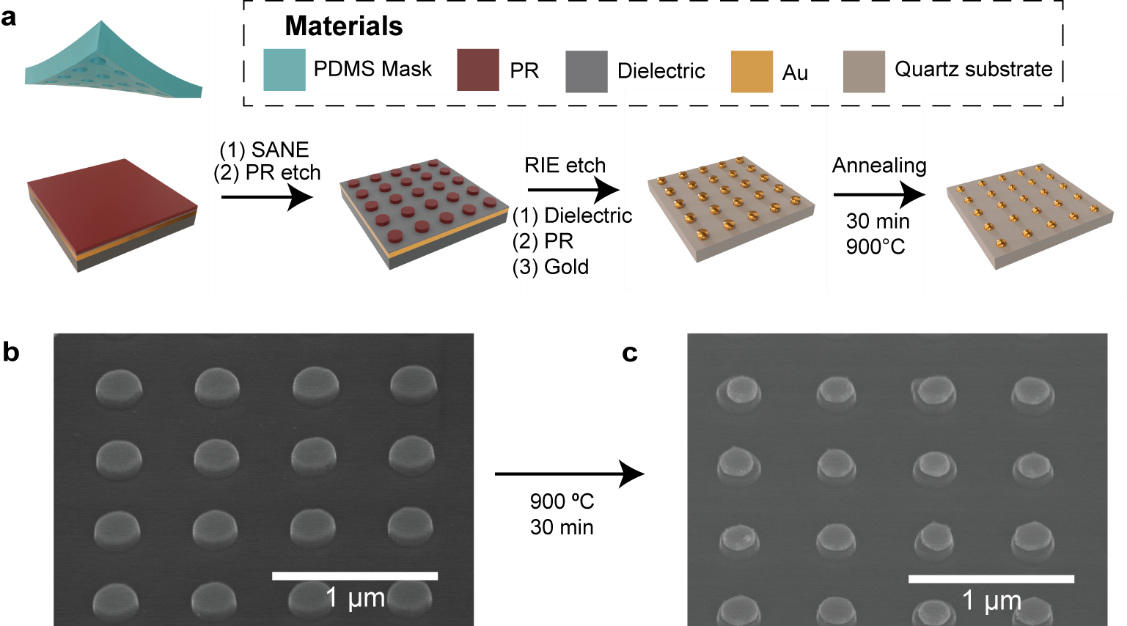


**Figure S1: Metal etching and thermal annealing for fabricating arrays of hemispherical nanoparticles. (a)** Fabrication scheme. Tilted (45°) SEM images of **(b)** as-fabricated cylindrical gold nanoparticles and **(c)** hemispherical nanoparticle arrays after annealing.

# Cell shape is less regular on nanoparticle arrays than on glass

Cells grown on nanoparticle arrays (**Figure S2a**) show very distinct morphologies from cells grown on glass (**Figure S2b**). Notably, the cells on arrays are significantly more elongated and show more large protrusions than their equivalents on glass. To quantify this difference, we measured the dimensionless shape parameter of cells under both conditions (**Figure S2c**). The shape parameter ρ is defined by the following equation: $\rho= \frac{P}{\sqrt{A}}$ where P is the perimeter of the cell and A is the area. This shape parameter has a minimum value of 3.54 for the case of a perfectly circular cell and is larger as the regularity of the cell shape decreases.^[S2]^ In models, a higher shape parameter is asociated with higher cell motility and less propensity for jamming.^[S3]^ In our case, we observed that the shape parameter is considerably lower on glass than on arrays, confirming our observation that the regularity of shape is affected by the array patterns.

**
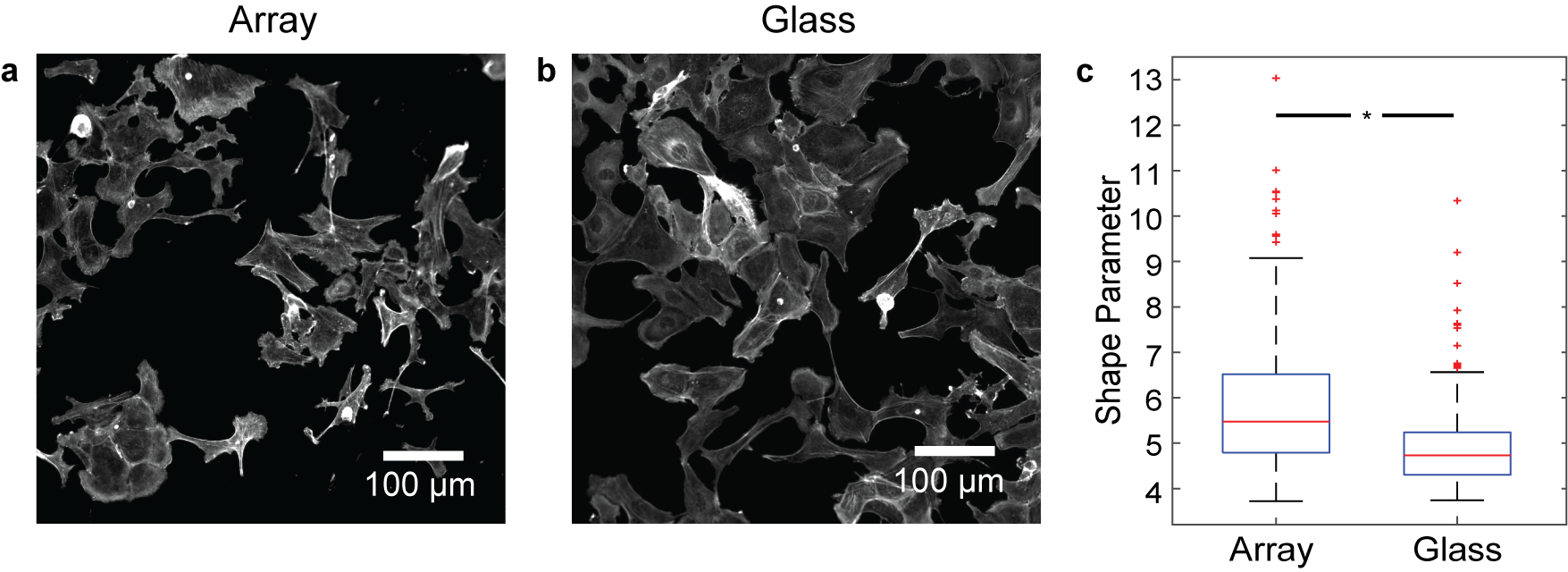
**

**Figure S2: Cells on arrays have less regular shapes than cells on glass. (a)** Cells on gold nanoparticle arrays are elongated and tend to have numerous large protrusions. **(b)** Cells grown on glass substrates are rounder and have fewer protrusions. **(c)** Measurement of the shape parameter reveals that cells on arrays are much less regular in shape than cells on glass. * $p< {10}^{-12}$ Significance computed using one-way ANOVA.

# Correlation between actin and Arp3 is increased on nanoparticle arrays

For cells on nanoparticle arrays, actin and Arp3 puncta can both be observed on array sites. To test whether the different proteins also associate with each other, we measured the Pearson’s correlation coefficient using the Coloc 2 plugin that comes with the FIJI distribution of ImageJ software. Because the Arp3 channel displays significant background noise, the correlation between entire fields of view are low irrespective of any actual correspondence between that channel and actin. As such, we restricted measurements to regions of interest that displayed a high density of puncta. **Figure S3** shows an increase in the correlation between actin and Arp3 between glass and array conditions.

**
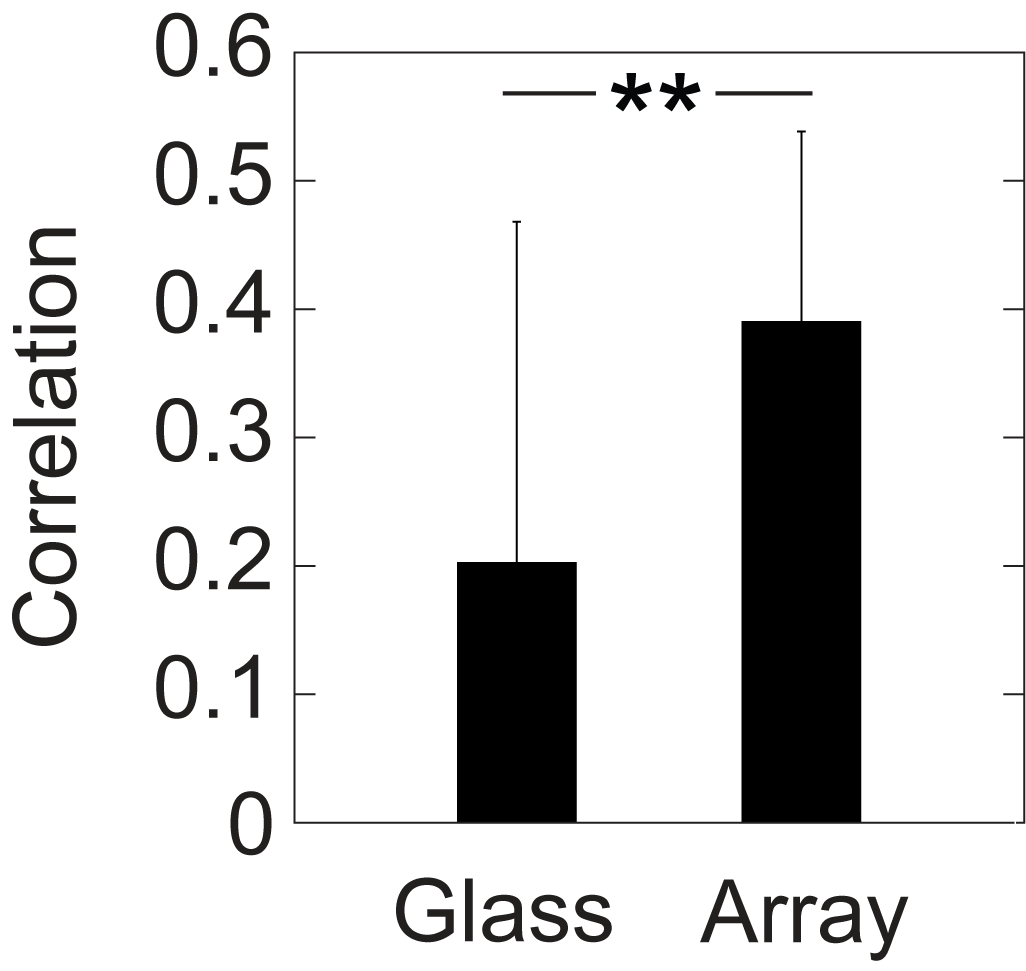
**

**Figure S3: Pearson’s correlation coefficient between the actin and Arp3 channels increases around array regions with high density of puncta.** **p < 0.005. Significance computed using one-way ANOVA.

# Cell shape and curvature sensing remain consistent on arrays with *a*_0_ = 600 nm

Since arrays with *a*_0_ = 1200 nm can induce curvature sensing and focal adhesion changes on cells, we tested whether arrays with *a*_0_ = 600 nm could promote similar effects. We cultured cells on 600 nm arrays with no drug treatment and immunostained for actin, Arp3, and paxillin (**Figure S4**). We observed no major differences between *a*_0_ = 1200 nm and *a*_0_ = 600 nm arrays; in both cases, there were sporadic actin puncta colocalized with Arp3 puncta, and focal adhesions were reduced in size compared to the glass case.

**
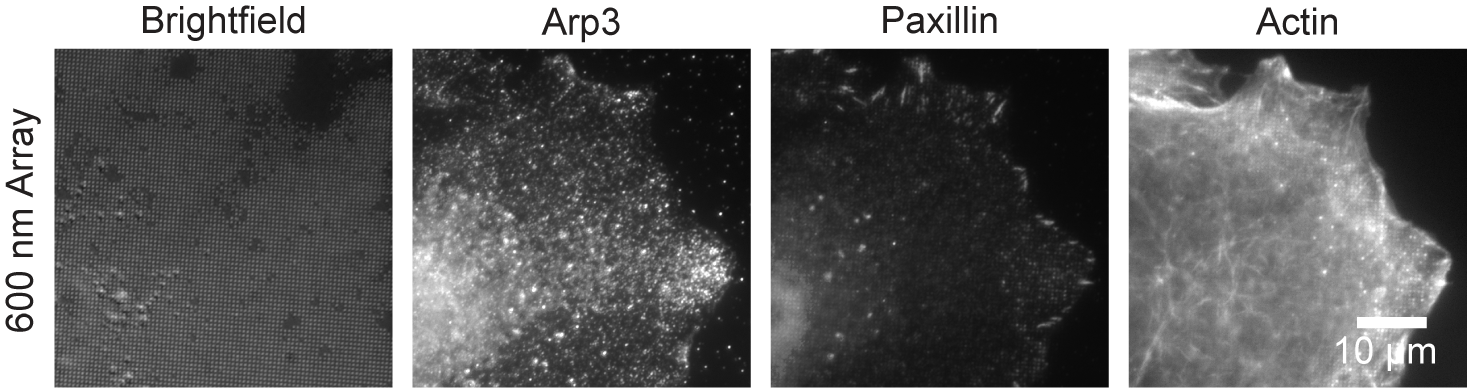
**

**Figure S4: Arrays with a pitch of 600 nm induce cytoskeletal reorganization and reduce focal adhesion size compared to glass.**

# Periodicity of actin puncta decreases with increasing tension

Since the arrays used for cell culture are periodic with a pitch of 1200 nm, more actin puncta colocalizing with array sites will result in a periodic pattern with higher contrast. This periodicity can be estimated visually using the fast Fourier transform (FFT) of the relevant channel. We applied FFTs to each actin channel image across different conditions (n = 40 for each condition) and then averaged the FFT images to determine the typical periodicity of actin puncta under each condition (**Figure S5**). Periodicity can be resolved under low-tension conditions. Under control conditions, there is some faint periodic structuring, which is consistent with weak periodicity under these conditions. When cells are treated with RhoA Act II, the FFT displays minimal periodicity.

**
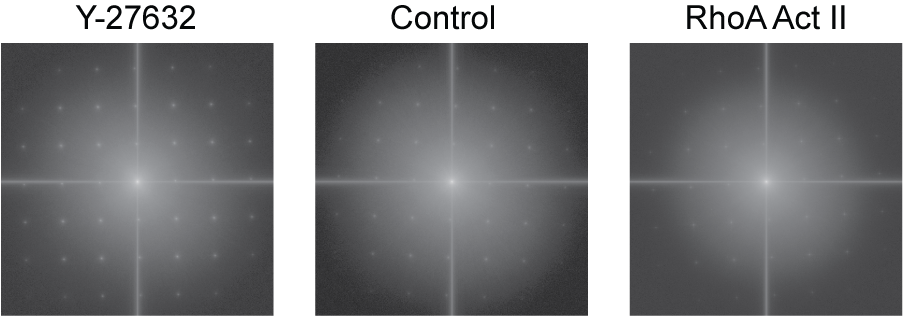
**

**Figure S5: Cytoskeletal tension reduces array-colocalized branched actin networks in favor of stress fiber formation.**

# Treatment with Y-27632 increases correlation between actin and nanoparticle signals

To quantify the relationship between cytoskeletal reorganization and nanoparticles, we measured the correlation between the actin channel and the brightfield channel (nanoparticles appear as diffraction-limited dark spots). We then processed and analyzed the images to find the Pearson’s correlation between the actin channel and the barightfield channel (Supplementary Methods). **Figure S6** shows the results of this quantification, where most noticeably, cells treated with Y-27632 displayed a significantly higher correlation than either control or Rho Act II-treated cells.

**
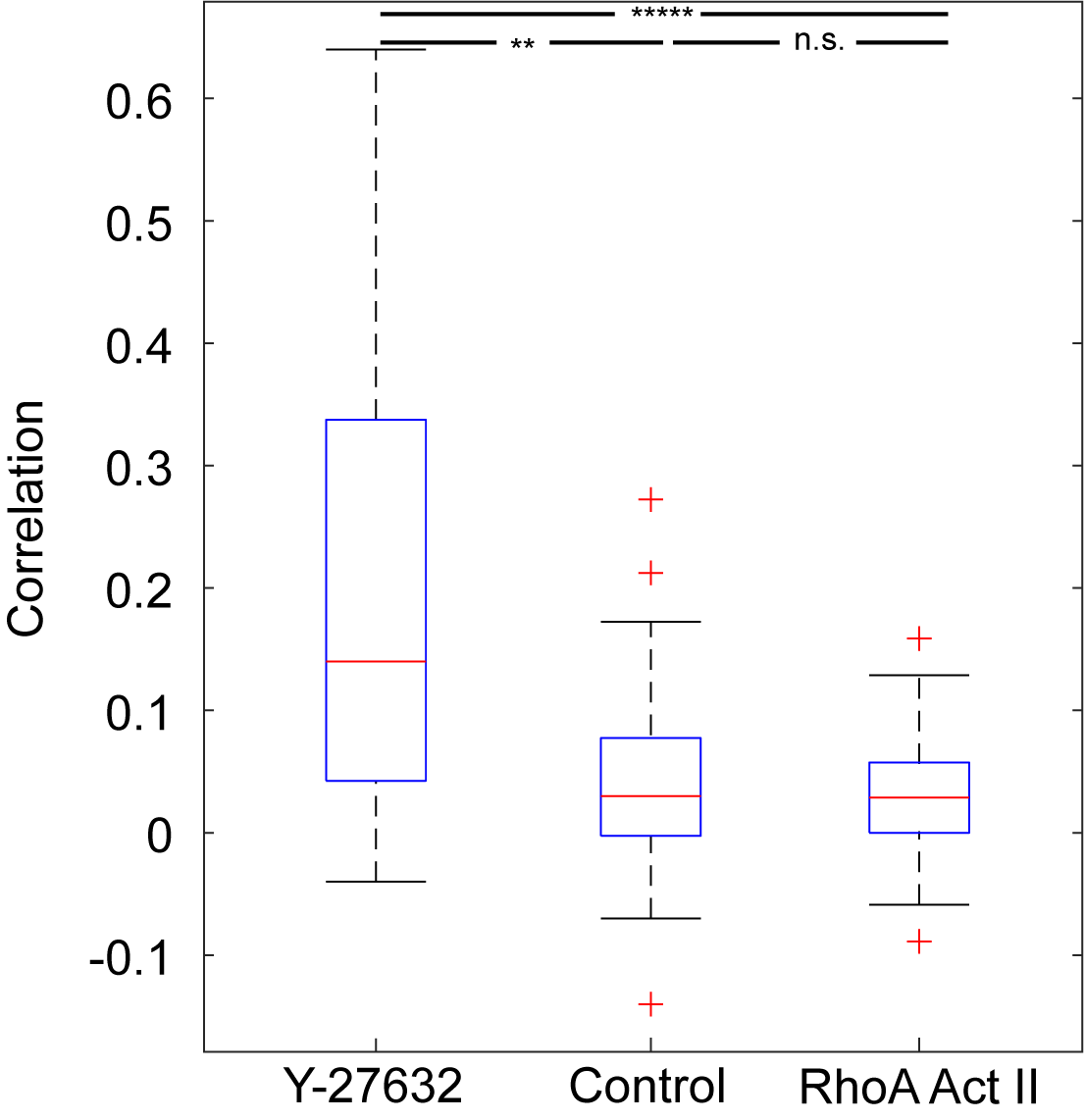
**

**Figure S6: Reducing cytoskeletal tension through Y-27632 treatment is associated with significantly higher correlation between the actin and brightfield channels,** indicating a higher degree of colocalization between actin and nanoparticle sites. **, ***** $p< {10}^{-2}, p< {10}^{-5}$ respectively. Significance computed using one-way ANOVA.

# Stress fiber coverage shows increased tension with increased RhoA activity

Quantitative techniques for measuring cell tension, such as traction force microscopy, require deformable substrates and are incompatible with our topological structures. However, cytoskeletal tension may be estimated by examining the prevalence and thickness of stress fibers within cells^[S4]^ (**Figure S7**). With the ROCK inhibitor Y-27632, stress fibers are absent, indicating that tension is very low. Under control conditions, there are a few scattered stress fibers, which are quite thin. With RhoA Act II treatment, the stress fibers are large and numerous, indicating increased tension.


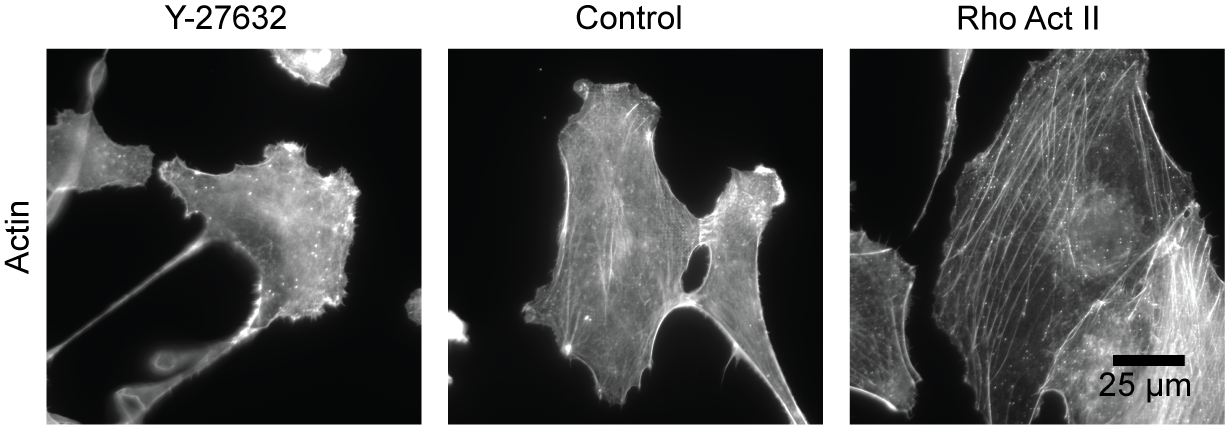


**Figure S7: Pharmacological treatments change the tension levels within cells on arrays.**

# Focal adhesions of cells on glass adopt large plaque morphologies

Cells grown on glass and other hard substrates display a few common focal adhesion characteristics in the absence of any other stimuli.^[S5,S6]^ **Figure S8** shows focal adhesions are large and display an elongated ellipsoid morphology. Also, the focal adhesions are located at the ends of stress fibers. Since stress fibers are more common on hard substrates than soft substrates, focal adhesions are also more prevalent.^[S7]^

**
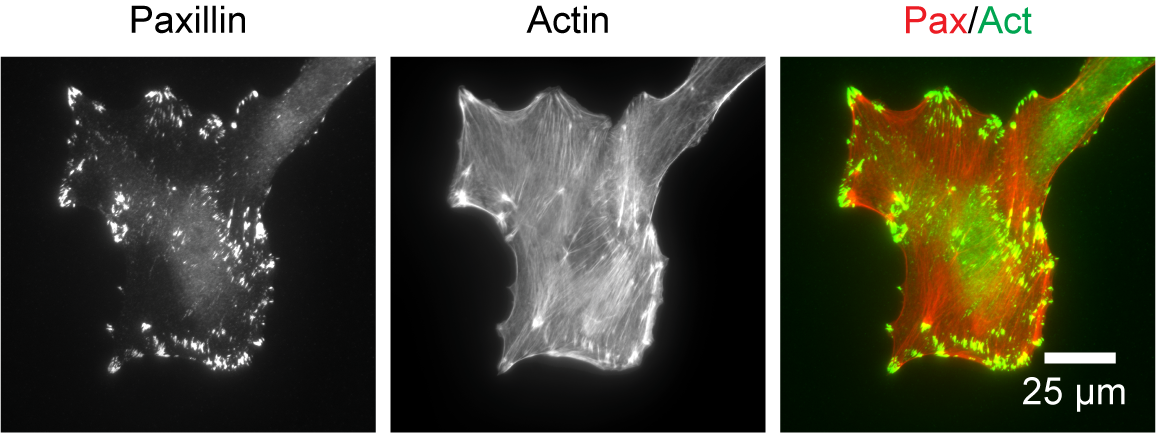
**

**Figure S8: Cells grown on glass display characteristic focal adhesion shape and location.**

# Focal adhesions on arrays are larger with higher RhoA activity

Since topographical features reduce the size of focal adhesions, we tested whether different levels of RhoA/ROCK activity affected how focal adhesions responded to array sites. We measured the size of focal adhesions using ImageJ (Supplementary Methods). **Figure S9** shows while there is no significant difference in focal adhesion size between control cells and those treated with Y-27632, focal adhesions in cells treated with RhoA Act II are considerably larger.


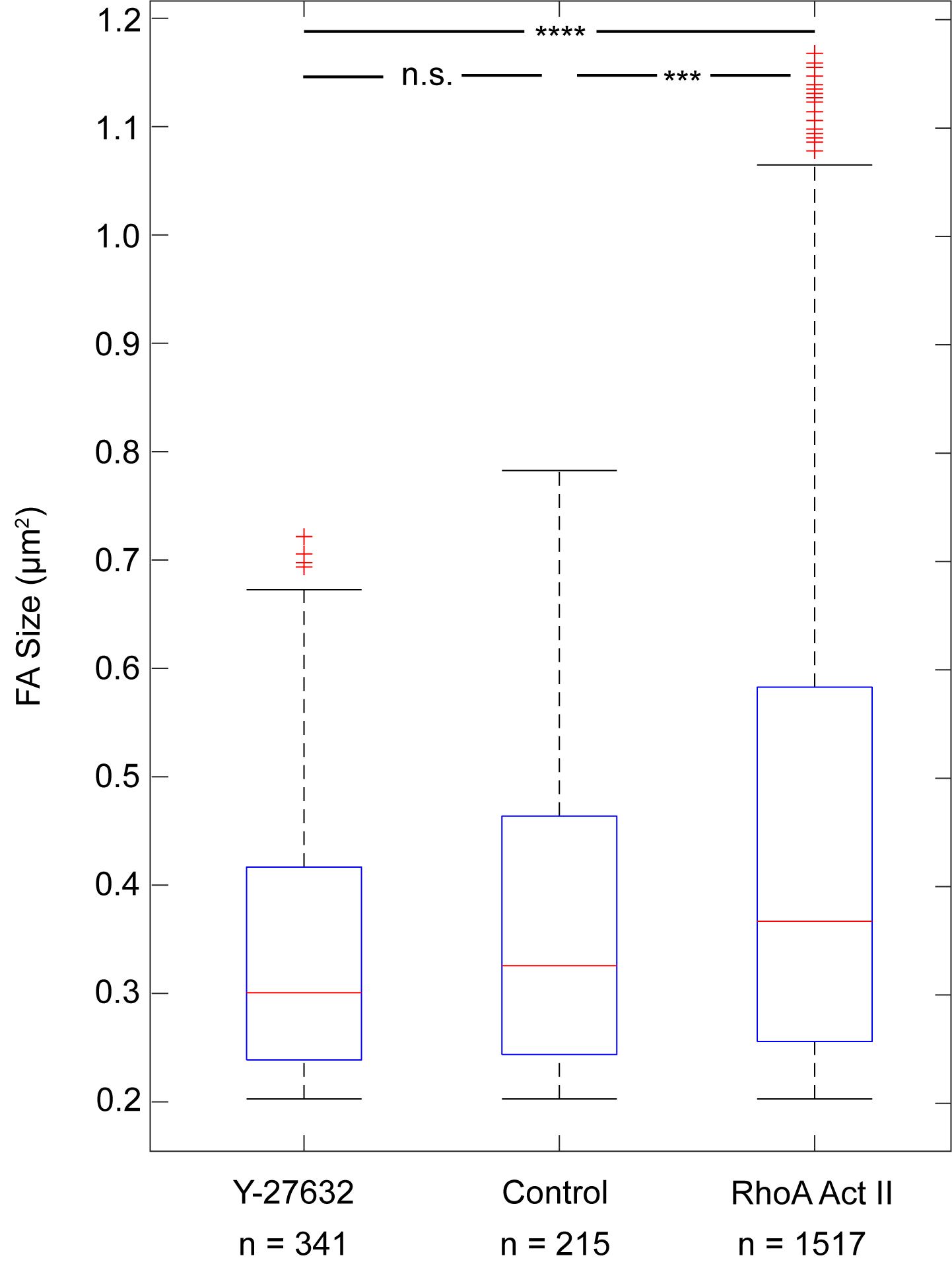


**Figure S9: Cells treated with RhoA Act II display significantly larger focal adhesions** than control or Y-27632-treated cells. *** $p<1\times{10}^{-3}$, **** $p<1\times{10}^{-4}.$ Significance computed using one-way ANOVA.

# Contiguous patches of array-patterned paxillin are larger with reduced RhoA activity

While all pharmacological conditions produced some patches where paxillin puncta had a one-to-one correspondence with the underlying array structure, the patches were of different sizes. To quantify this observation, we generated masks corresponding to each patch in ImageJ and used the Analyze Particles function to measure the size. **Figure S10** indicates that the patches tend to be equivalent in size for RhoA Act II and the control case but are significantly larger when cells undergo Y-27632 treatment. Hence, the formation of array-associated focal adhesions depends on cytoskeletal tension that we associate with membrane conformality around the gold nanoparticles.

**
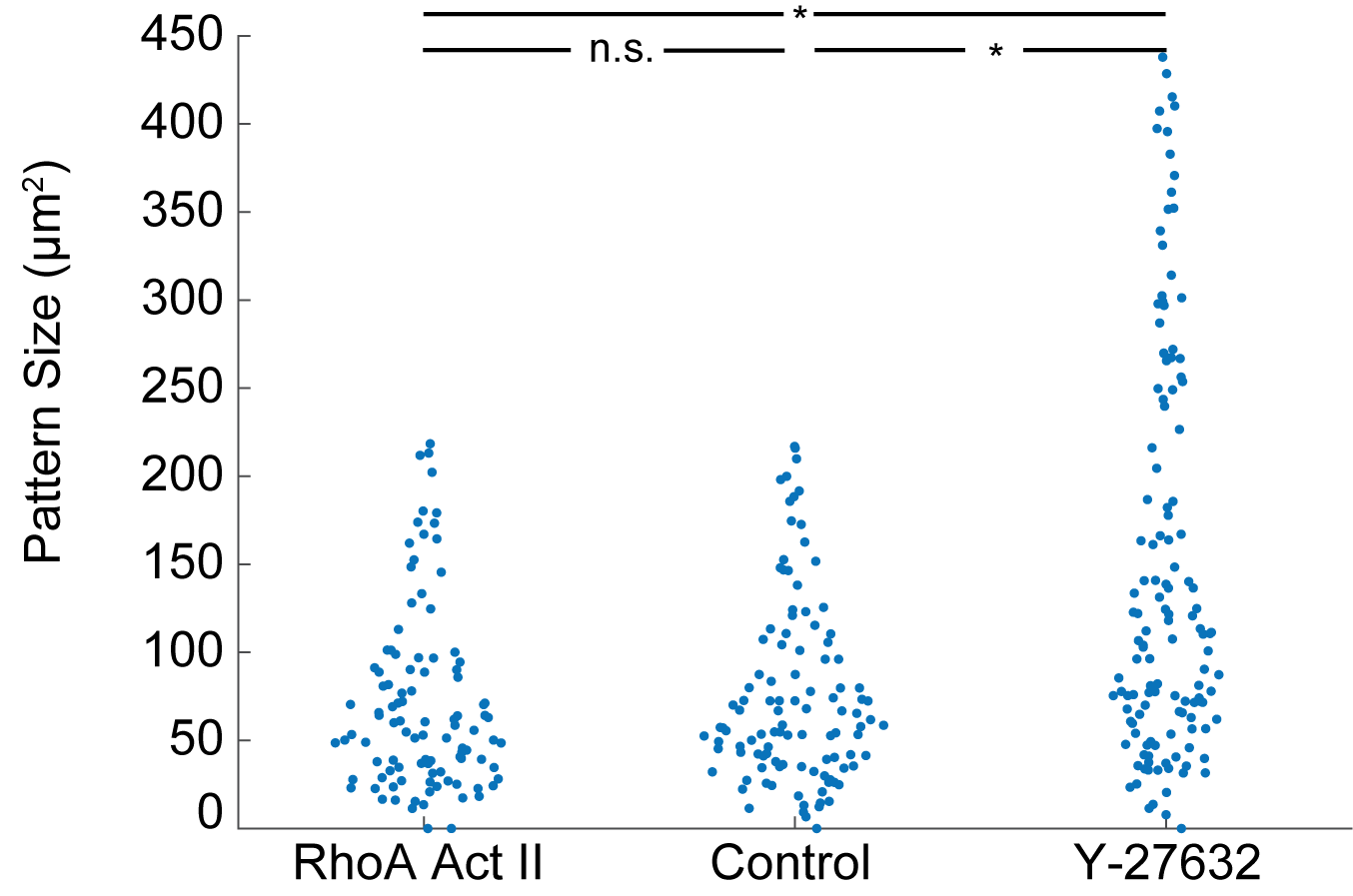
**

**Figure S10: Patches of array-patterned paxillin are larger upon Y-27632 treatment than in other conditions.** * $p<1\times{10}^{-8}$. Significance computed using one-way ANOVA.

# Treatment with Y-27632 or RhoA Act II does not affect cell shape

Since RhoA is responsible for regulating contractility in cells, we cannot exclude the possibility that Y-27632 and RhoA Act II could affect factors such as cell shape parameter, which would carry implications for cell motility. Hence, we measured the shape parameter of cells treated with both of the drugs tested and found no significant differences between the treatment conditions (**Figure S11**). These results show that, while the mean shape parameter is slightly different between treatments, the cell-to-cell variability within each treatment is larger than the differences between treatments.

**
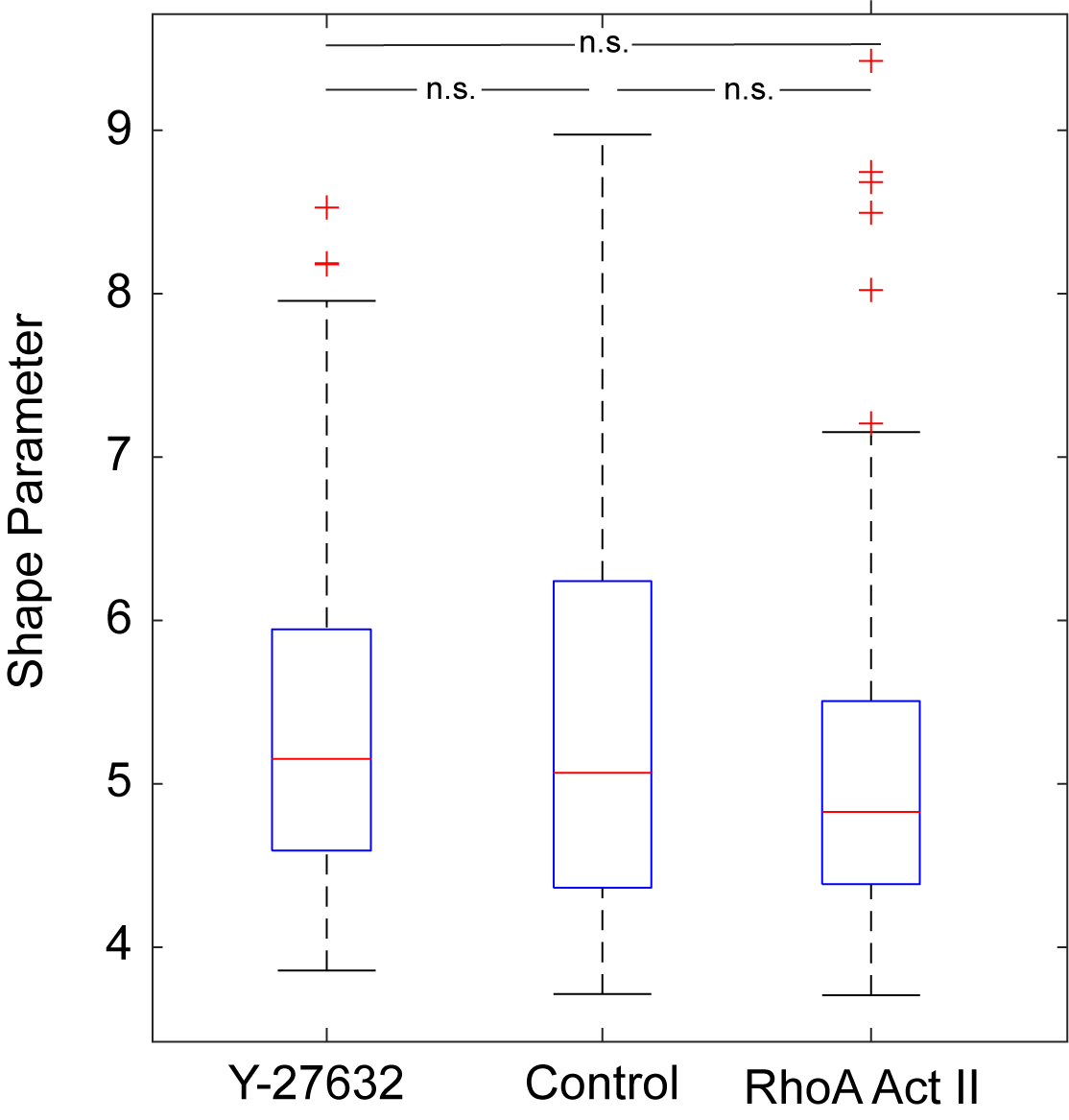
**

**Figure S11: Shape parameter is not significantly different when cells on arrays are treated with Y-27632, kept under control conditions, or treated with RhoAct II.** Significance computed using one-way ANOVA.

# Supporting Information References

[S1] A. E. Minuti, L. Labusca, D.-D. Herea, G. Stoian, H. Chiriac, N. Lupu, *IJMS* **2022**, *24*, 430.

[S2] D. Bi, J. H. Lopez, J. M. Schwarz, M. L. Manning, *Nature Phys* **2015**, *11*, 1074.

[S3] D. Bi, X. Yang, M. C. Marchetti, M. L. Manning, *Phys. Rev. X* **2016**, *6*, 021011.

[S4] N. D. Bade, R. D. Kamien, R. K. Assoian, K. J. Stebe, *Sci. Adv.* **2017**, *3*, e1700150.

[S5] P. Kanchanawong, G. Shtengel, A. M. Pasapera, E. B. Ramko, M. W. Davidson, H. F. Hess, C. M. Waterman, *Nature* **2010**, *468*, 580.

[S6] P. W. Oakes, Y. Beckham, J. Stricker, M. L. Gardel, *J Cell Biol* **2012**, *196*, 363.

[S7] P. Hotulainen, P. Lappalainen, *J Cell Biol* **2006**, *173*, 383.
